# Valproic acid ameliorates cauda equina injury by suppressing HDAC2‐mediated ferroptosis

**DOI:** 10.1111/cns.14524

**Published:** 2023-12-17

**Authors:** Qingjie Kong, Fudong Li, Kaiqiang Sun, Xiaofei Sun, Jun Ma

**Affiliations:** ^1^ Department of Orthopedics Shanghai General Hospital, Shanghai Jiao Tong University School of Medicine Shanghai China; ^2^ National Key Laboratory of Medical Immunology & Institute of Immunology Second Military Medical University Shanghai China; ^3^ Department of Orthopedic Surgery Spine Center, Shanghai Changzheng Hospital, Second Military Medical University Shanghai China

**Keywords:** cauda equina injury, ferroptosis, HDAC2, neuroinflammation, valproic acid

## Abstract

**Introduction:**

Persistent neuroinflammatory response after cauda equina injury (CEI) lowers nociceptor firing thresholds, accompanied by pathological pain and decreasing extremity dysfunction. Histone deacetylation has been considered a key regulator of immunity, inflammation, and neurological dysfunction. Our previous study suggested that valproic acid (VPA), a histone deacetylase inhibitor, exhibited neuroprotective effects in rat models of CEI, although the underlying mechanism remains elusive.

**Methods:**

The cauda equina compression surgery was performed to establish the CEI model. The Basso, Beattie, Bresnahan score, and the von Frey filament test were carried out to measure the animal behavior. Immunofluorescence staining of myelin basic protein and GPX4 was carried out. In addition, transmission electron microscope analysis was used to assess the effect of VPA on the morphological changes of mitochondria. RNA‐sequencing was conducted to clarify the underlying mechanism of VPA on CEI protection.

**Results:**

In this current study, we revealed that the expression level of HDAC1 and HDAC2 was elevated after cauda equina compression model but was reversed by VPA treatment. Meanwhile, HDAC2 knockdown resulted in the improvement of motor functions and pathologic pain, similar to treatment with VPA. Histology analysis also showed that knockdown of histone deacetylase (HDAC)‐2, but not HDAC1, remarkably alleviated cauda equina injury and demyelinating lesions. The potential mechanism may be related to lowering oxidative stress and inflammatory response in the injured region. Notably, the transcriptome sequencing indicated that the therapeutic effect of VPA may depend on HDAC2‐mediated ferroptosis. Ferroptosis‐related genes were analyzed in vivo and DRG cells further validated the reliability of RNA‐sequencing results, suggesting HDAC2‐H4K12ac axis participated in epigenetic modulation of ferroptosis‐related genes.

**Conclusion:**

HDAC2 is critically involved in the ferroptosis and neuroinflammation in cauda equina injury, and VPA ameliorated cauda equina injury by suppressing HDAC2‐mediated ferroptosis.

## Graphical abstract

Cauda equina injury (CEI) leads to ferroptosis in mice by raising the expression level of HDAC2, resulting in an increase in MDA and ROS. Ferroptosis in CEI is ameliorated by VPA treatment via suppressing HDAC2.

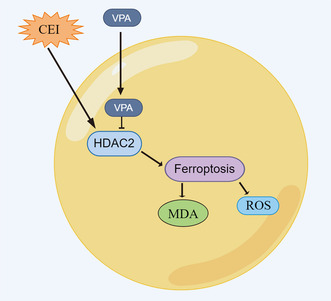

## 1 INTRODUCTION

Cauda equina syndrome (CES) is a common clinical disorder of intraspinal sacral and lumbar nerve root caused by spinal stenosis, lumbar disc herniation, trauma, tumor, and spinal epidural hematoma.^1^, ^2^ As a bridge between spinal cord neurons and dorsal root ganglion (DRG) pseudounipolar neurons, the cauda equina has only one layer of endoneurial tissue, predisposing it to mechanical and inflammatory damage.^3^, ^4^ After mechanical injury, massive infiltration of immune cells and release of inflammatory factors enhanced the neuroinflammatory response. Cascade amplification of the inflammatory response causes neuronal death and reduces the thresholds of A‐σ and C‐fiber nociceptors, resulting in motor dysfunction and pathological pain.^5^ Surgical treatment can only spare the mechanical compression, but with other complications, such as sellar sensory disorders, intestinal, and sexual dysfunction.^6^ Therefore, there is an urgent demand to develop an effective therapy to treat CES. Our previous study demonstrated that valproic acid (VPA) could alleviate cauda equina injury (CEI),^7^ reduce apoptotic cells, and improve motor recovery, suggesting a neuroprotective effect in acute CEI. However, the underlying mechanisms need to be further explored.

VPA, a short‐chain fatty acid histone deacetylase (HDAC) inhibitor, exerts antiepileptic and anti‐lymphoid properties in the previous study.^8^ Ryan W. Logan reported that VPA improved mania‐like behaviors in mice by preferential‐targeting HDAC2.^9^ Francesca Paino et al. reported that VPA downregulated osteocalcin gene expression and enhanced osteoblast differentiation, which was strongly correlated with inhibition of HDAC2.^10^ All these studies indicated that HDAC2 is an important molecule for VPA function. HDAC2, a member of the histone deacetylase family, removes acetyl groups from histone tails and therefore is considered a transcriptional corepressor.^11^ Increasing studies suggested that HDAC2 contributed to the development of many diseases by promoting oxidative stress and inflammatory response.^12^, ^13^, ^14^ Sun et al. found that LPS induced neuroinflammation by upregulating HDAC2 in the dorsal hippocampal and inhibiting HDAC2 could reduce microglial activation.^15^ Jiao et al. reported that HDAC2 inhibitor, CAY10683, could alleviate LPS‐induced neuroinflammation by inhibiting TLR4/NF‐κB signaling pathway.^16^ Whether VPA improves cauda equina injury by regulating HDAC2‐mediated mechanisms remains to be explored.

Ferroptosis is a kind of iron‐mediated cell death. Unlike other types of cell death, such as necroptosis and apoptosis, ferroptosis does not involve in caspase protein activation.^17^, ^18^ It is distinguished by increasing lipid peroxidation products and reactive oxygen species (ROS). Ferroptosis has been reported to contribute to the onset and development of cancer,^19^ inflammation‐related diseases,^20^ and neurodegenerative diseases.^21^ Several studies have demonstrated that neuroinflammation contributes to iron accumulation in the brain, which results in the death of neurons. ACSL4 exacerbates ischemic stroke by promoting ferroptosis‐induced brain injury and neuroinflammation.^22^ William Sealy Hambright reported that ablation of ferroptosis regulator, glutathione peroxidase 4, in forebrain neurons promoted cognitive impairment and neurodegeneration.^23^ Based on these studies, we hypothesized that ferroptosis may participate in the function of VPA in improving CEI.

This study aims to explore the roles of HDAC2 and VPA treatment in regulating ferroptosis and neuroinflammation in cauda equina injury through in vivo and in vitro experiments, and this study provides a new therapeutic target and a novel experimental basis for the protection against CEI.

## 2 MATERIALS AND METHODS

### 2.1 Experiment design

A randomized animal experiment approach was adopted. Rats were randomly allocated to five groups (sham, model control, model with VPA therapy, model with HDAC1‐KD, and model with HDAC2‐KD, *n* = 10). As in our previous study,^7^ silicon compression was used to induce cauda equina injury in rats, and an injection of VPA (300 mg/kg in saline) was given 2 h before surgery. HDAC1‐KD and HDAC2‐KD were established 7 days before the cauda equina compression (CEC) surgery as the previous study.^24^ After surgery, the VPA group received further VPA injections twice daily for 1 week. Neurobehavioral tests were performed on rats for weeks. Histopathologic investigations were then performed on the postoperative seventh day.

### 2.2 Animals and treatment

Male Sprague–Dawley rats (weighing approximately 250 g) were purchased from Slack Company (shanghai). All the animal experiments used in this study were approved by the Animal Ethics Committee of Shanghai Changzheng Hospital (no. 2021SL097). The rats were kept in a 12:12 light/dark cycle, with unrestricted food and water access. They were acclimatized for 1 week before the experiment and fasted for 12 h before surgery.

For lentivirus delivery, Lv‐shHDAC1 and Lv‐shHDAC2 were purchased from Genechem (shanghai, China). Rats received Lv‐shHDAC1 and Lv‐shHDAC2 (10 μL, 1 × 10^9^ TU/mL) via intrathecal catheter 7 days before the CEC surgery.

For the CEC model, the CEC rat model was established as our previous study reported. Briefly, the rats were anesthetized and placed in a prone position. An incision was made at the L4 to S2 level to expose the L4 to L5 lamina. Then, the ligamentum flavum between L5 and L6 was removed. A 10‐mm‐long, 1‐mm‐wide, and 1‐mm‐thick silicone block was inserted into the epidural space under the L5 to L6 vertebrae. Sham‐operated rats underwent the same surgery procedure without the silicon insertion.

On the seventh day following surgery, five rats from each group were sacrificed for experimental examinations. The DRGs, cauda equina (L5–L6) were dissected. Some tissues were fixed with 4% PFA, embedded in paraffin, and cut into 5‐μm sections. The other part was flash‐frozen in liquid nitrogen and stored in a −80 refrigerator for subsequent experiments.

### 2.3 Behavioral tests

Basso, Beattie, Bresnahan (BBB): the BBB test was designed to evaluate the recovery of motor function of hindlimb following CEC. After baseline testing at 2 h before the surgery, the rats were tested on postoperative days 3, 7, 14, 21, and 28. The main procedures of BBB locomotor rating scale were carried out according to the previous report.^25^

Mechanical hyperalgesia: the von Frey filament test (Aesthesio, RWD) was used to measure the mechanical hyperalgesia. Before testing, rats were placed in elevated plastic boxes and acclimated to the testing cage for 30 min. The plastic tip was then applied perpendicularly to the medial plantar surface of right hind paw until a clear withdrawal was observed. Mechanical thresholds were recorded when withdrawal, flinching, paw licking, or toe spreading was observed, and the rats were tested on postoperative days 21, 28, and 42.

Rats were recorded in an open field and two independent observers were unaware of the experimental procedures.

### 2.4 Hematoxylin and eosin staining

Hematoxylin and eosin staining (H&E) was performed according to previous studies. In brief, the sections of each group were heated at 60°C for 30 min followed by immersion in xylene I and II for 30 min and placed in gradient alcohol solutions for 5 min. The slices were then stained with hematoxylin for 2 min, washed twice with distilled water, differentiated with 1% hydrochloric acid, rinsed twice with distilled water, and stained with eosin for 2 min.

### 2.5 Luxol fast blue staining

The LFB staining was done according to our previous study. Briefly, the sections of each group were deparaffinized and hydrated in 95% ethyl alcohol for 1 h, then placed into Luxol Fast Blue solution overnight. The next day the sections were rinsed with 95% ethyl alcohol and distilled water, differentiated in the lithium carbonate solution for 30 s, differentiated in the 70% ethyl alcohol for 30 s, and then rinsed with distilled water two times. The above two steps were repeated until the gray and white matter separated. Then the sections were stained with an eosin solution and sealed with neutral gum.

### 2.6 Immunofluorescence staining (IFC)

IFC staining was performed as reported previously. Briefly, the frozen tissues were cut into 5‐um sections and incubated with anti‐GPX4 antibody (1:200, ab125066, Abcam) overnight at 4C. The next day, sections were washed by PBS for three times, then incubated in DyLight 488 AffiniPure Goat‐anti‐rabbit IgG (1:400, Abcam) for 1 h at RT.

### 2.7 Reactive oxygen species (ROS), MDA, and GSH content assay

The level of ROS, the GSH, and MDA content in cell lysis and tissue homogenates was assessed by a tissue reactive oxygen species (ROS) Detection Kit (Bestbio China), a Glutathione Assay Kit (Sigma, CS0260) and lipid peroxidation kit (Sigma, MAK085) in accordance with the standard protocol, respectively.

### 2.8 Dihydroethidium (DHE) staining

Tissue sections were incubated with 2 μmol/L fluorescent dye DHE (Beyotime, China) for 30 min in a dark, humid environment at 37°C.

### 2.9 Chromatin immunoprecipitation (ChIP)

Cauda equina tissues were chemically cross‐linked by 11% formaldehyde solution at RT for 15 min, resuspended, lysed in lysis buffers, and sonicated to solubilize and shear cross‐linked DNA. The sample was sonicated at 4°C. The sample extract was incubated with 100 mL of CHIP‐Grade Protein G Magnetic Beads (GST 9006) that have been pretreated with 10 mg of IgG, HDAC1, and HDAC2 antibody. After being washed with RIPA buffer five times and with 10 mM Tris–HCl (with 1 mM EDTA and 50 nM NaCl). The extracted DNA was eluted from the beads overnight at 65°C. Then the DNA was purified by RNaseA, proteinase K, and 1 mM Tris–HCl (with 0.5 mM EDTA). Then the Purified DNA samples were normalized and subjected to PCR analysis.

### 2.10 Cell

L4/L5 DRG was first homogenized in cold calcium‐ and magnesium‐free Hanks' balanced salt solution. Homogenize the DRG tissue in the Dounce homogenizer 30 times. The homogenized tissue suspension is filtered through a 70 m nylon cell strainer. The homogenizer was washed with ice‐cold HBSS, and then the homogenizer with 1.5 mL of equilibrated ice‐cold isotonic density gradient medium. Then the cell mixture was centrifuged at 800 × *g* 4°C for 15 min, and the cells were resuspended in PBS containing 5% FBS for enrichment.

For HDAC2 overexpression and knockdown, lentivirus production of HDAC2 and si‐HDAC2 was obtained from Genechem (shanghai, China) and transfected DRG as directed. Cells were grown for an extra 24 h after transfection and then retrieved for the following experiment.

For inhibitor treatment, DRG cells were treated with 1 μM of Ferrostatin‐1, 10 μM of ZVAD‐FMK, or 10 μM of necrostatin‐1.

### 2.11 Cell counting kit‐8 (CCK‐8) assay

DRG cell viability was analyzed by CCK‐8 array (Beyotime, China). DRG cells with different treatments were seeded onto 96‐well plates (1 × 10^4^). After 24 h, CCK‐8 reagent (10 μL) was added to each well for an additional 4 h at 37°C, and the absorbance at 450 nm of each well was assayed using a microplate reader (BioTek Instruments).

### 2.12 Fluorescein diacetate (FDA) staining

DRG cells were incubated with 5 μL of Fluorescein diacetate solution for 20 min at 37°C, then cells images were obtained using a fluorescence microscope (Olympus Corporation, Japan).

### 2.13 Western blotting

Western blotting was performed as reported in the previous study.^26^ Briefly, the protein of tissue or cells was extracted by RIPA Buffer (Beyotime), and protein concentration was assessed by a BCA kit (Beyotime). Approximately 20 μg of each sample was uploaded to the SDS‐PAGE gel. The first antibody was used as followed: HDAC1(1:1000, ab109411, Abcam), HDAC2 (1:1000, ab32117, Abcam), p‐P65 (1:500, ab239882, Abcam), P65 (1:1000, ab19870, Abcam), p‐IκB(1:1000, ab133462, Abcam), IκB (1:1000, ab32518, Abcam), GPX4(1:1000, ab125066, Abcam), SLC7A11(1:500, ab238969, Abcam), Nrf2 (1:1000, ab92946, Abcam), FTH1(1:1000, ab183781, Abcam), PTGS2 (1:1000, ab179800, Abcam), Ac‐H3K14(1:2000, ab52946, Abcam), Ac‐H4K12(1:5000, ab177793, Abcam), and Actin (1:5000, ab8226, Abcam).

### 2.14 Quantitative real‐time PCR (qRT‐PCR)

The total RNA of tissue and cell was isolated with TRIzol (Sigma, USA) according to the instructions. First‐strand cDNA synthesis kit (Genenode, Beijing, China) was used to synthesize cDNA from total RNA (2 μg/sample), and then qRT‐qPCR was performed with SYBR Green PCR Mastermix (Solarbio, China). The foldchanges of RNA transcripts were calculated by the 2−^ΔΔCt^ method, and the β‐actin was used as a reference gene. The qPCR primer pairs are in Table 1.

**TABLE 1:** Primer for qRT‐PCR.

Primer	Sequence
IL‐1β	
Forward	CTACAATGAGCTGCGTGTGGC
Reverse	CAGGTCCAGACGCAGGATGGC
IL‐6	
Forward	CCGGAGAGGAGACTTCACAG
Reverse	ACAGTGCATCATCGCTGTTC
TNF‐α	
Forward	CAGATGGGCTGTACCTTATC
Reverse	GACTCCGTGATGTCTAAGTAC
HDAC2	
Forward	CTGCAGTTGCCCTTGATTGT
Reverse	CAGGCGCATGTGGTAACATT
β‐Actin	
Forward	GAAACAGCAATGGTCGGGAC
Reverse	AAGACACGGGTTCCATGGTG

### 2.15 Transmission electron microscopy (TEM)

DRG cells were treated with si‐HDAC2 for 24 h, followed by treatment with 10 μM of erastin for an additional 24 h. After 4 h of fixation with 2.5% glutaraldehyde, DRG cells were then fixed with 1% osmotic acid at RT for 120 min, dehydrated in an ethanol gradient, penetrated, and polymerized for 2 days at 60°C. 80 nm slices were produced and stained with 2% uranyl acetate and lead citrate saturated in aqueous solution. The image of DRG cell was taken by TEM (HT7800, Japan).

### 2.16 Statistical analysis

All statistical analyses were carried out by SPSS 21.0 software (IBM, USA). All data were subject to the Shapiro–Wilk test for normality analysis and all data exhibited normality. All the data were shown as the mean ± standard deviation (SD). The Student *t*‐test was used to establish the significance of two groups, and one‐way ANOVA followed by the Tukey–Kramer multiple comparisons test was used to determine the significance of more than two groups. A *p* < 0.05 was defined as a statistically significant difference.

## 3 RESULTS

### 3.1 VPA improved the neurological functions

**FIGURE 1:**
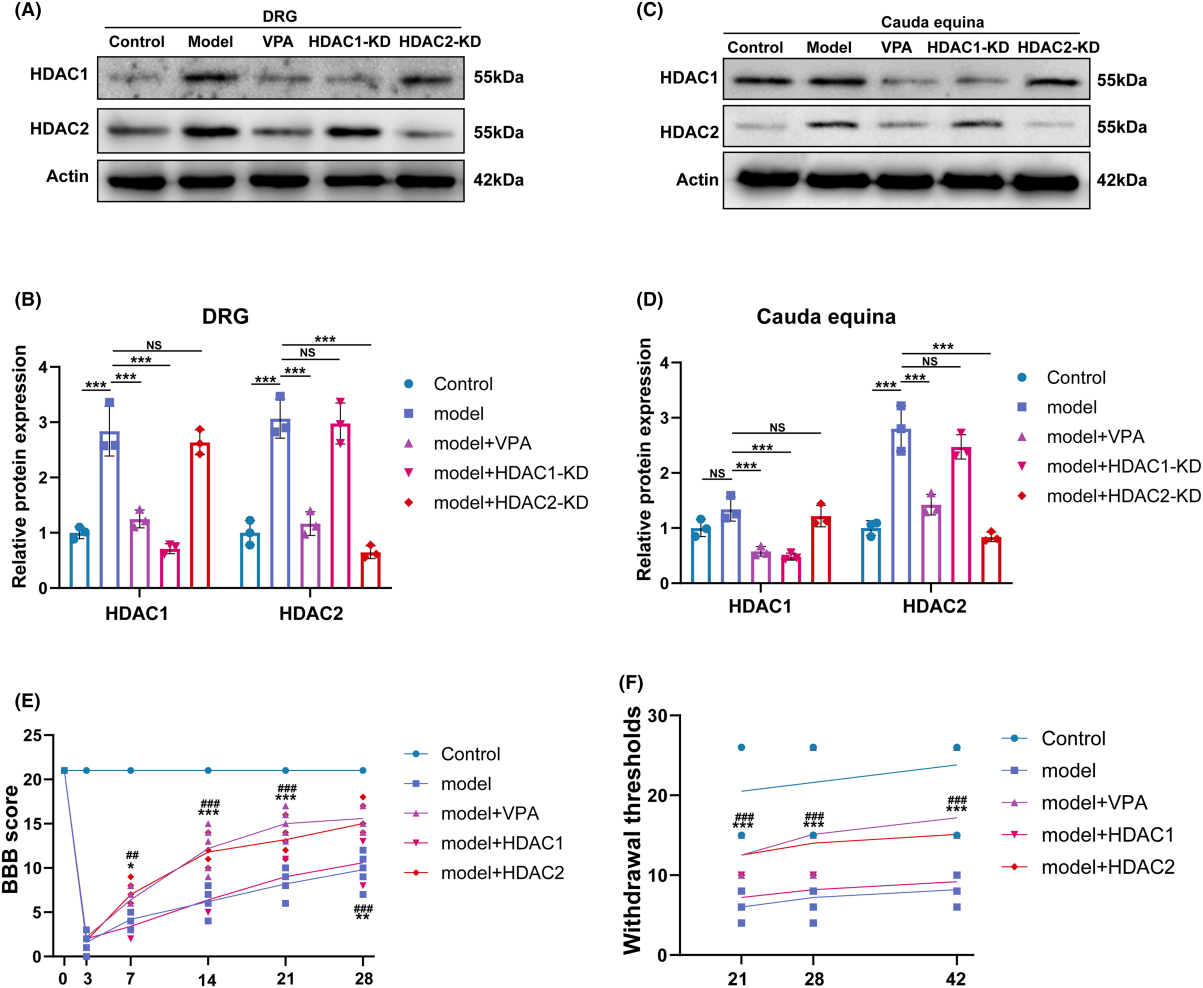
VPA improved the neurobehavioral depends on HDAC2 rather than HDAC1. (A, B) Western blotting and semi‐quantifications for HDAC1 and HDAC2 protein in DRG (*N* = 5). (C, D) Western blotting and semi‐quantifications for HDAC1 and HDAC2 protein in cauda equina (*N* = 5). (E, F) Statistical analysis of the BBB (*N* = 5) and thermal threshold (*N* = 10) of mice in different groups (“*”: model vs model+VPA; “^”: model vs model + HDAC1‐KD; “#”: model vs model + HDAC2‐KD.). Each data point was mean ± s.d. *p < 0.05; **p < 0.01; ***p < 0.001.

Our previous study confirmed that VPA had a neuroprotective effect in the rat CEI model. A large number of previous studies have confirmed that the function of VPA mainly depends on the inhibition of HDAC1 and HDAC2. To further explore whether the protective effect of VPA on CEI depends on the inhibition of HDAC1 and HDAC2, the expression of HDAC1 and HDAC2 was knocked down by lentivirus in the CEC rat model, and the efficiency of knockdown was confirmed by western blot of DRG neurons and cauda equina. As shown in Figure 1A–D, the expressions of HDAC1 and HDAC2 in DRG and cauda equina were upregulated in the CEC model, while the expressions were significantly restored after VPA treatment. Meanwhile, endogenous HDAC1 and HDAC2 expressions were downregulated by the lentivirus. To investigate the neuroprotective effects of VPA or HDAC‐KD, the motor function recovery was evaluated by visual observation according to the Basso, Beattie, Bresnahan (BBB) Locomotor Rating Scale, and the sensory recovery was estimated by the withdrawal of von Frey‐simulated hind limbs after CEI in rats. As indicated in Figure 1E, no evident difference was observed in BBB scores between HDAC1‐KD and CEC model groups. However, the BBB scores for VPA and HDAC2‐KD groups were significantly higher than that of the CEC model group at day 7 after CEC operation, indicating VPA treatment or HDAC2‐KD could drastically alleviate motor dysfunction. The mechanical allodynia test revealed a significantly higher mechanical threshold in VPA and HDAC2‐KD groups compared with the CEC model group on days 21 following CEI, and these differences persisted to day 42, indicating less tactile hypersensitivity. Although there were no obvious differences in mechanical threshold stimuli between the HDAC1‐KD and CEC model groups (Figure 1F). Combined with the results above, we reasoned that VPA and HDAC2‐KD could promote motor function recovery and reduce pathological pain after CEI.

### 3.2 VPA treatment and HDAC2‐KD improved the CEI

**FIGURE 2:**
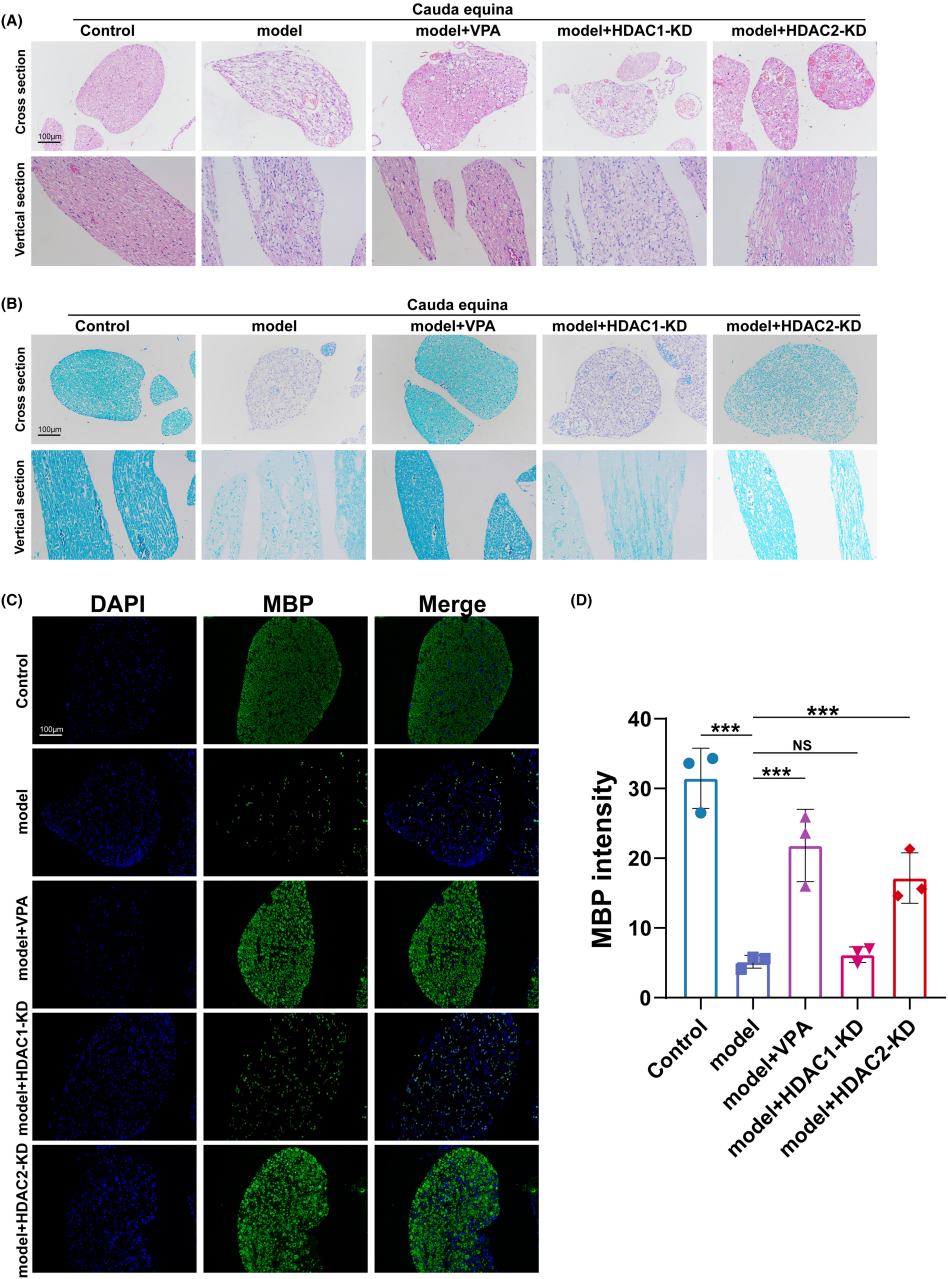
VPA treatment and HDAC2‐KD improved the cauda equina injury. (A) Hematoxylin & eosin (H&E) staining of mice in different groups (*N* = 5). (B) Luxol fast blue (LFB) staining of mice in different groups (*N* = 5). (C and D) Immunofluorescence and semi‐quantifications for myelin expression in different groups (*N* = 5). ns = no significant, **p < 0.01; ***p < 0.001.

To investigate the function of VPA, HDAC1‐KD, or HDAC2‐KD on the cauda equina injury and repair, the compressed part (L5‐L6) of the cauda equina was dissected on day 7 after surgery. The extent of CEC damage was assessed by H&E and LFB staining. As shown in Figure 2A,B H&E and LFB staining showed that the cauda equina in the model group showed obvious demyelination, myelin swelling, and neurodegeneration, while in the VPA and HDAC2‐KD groups, the myelin sheath and axon were more densely distributed and more intact in structure, and the cauda equina in the HDAC1‐KD group did not show such improvements.

**FIGURE 3:**
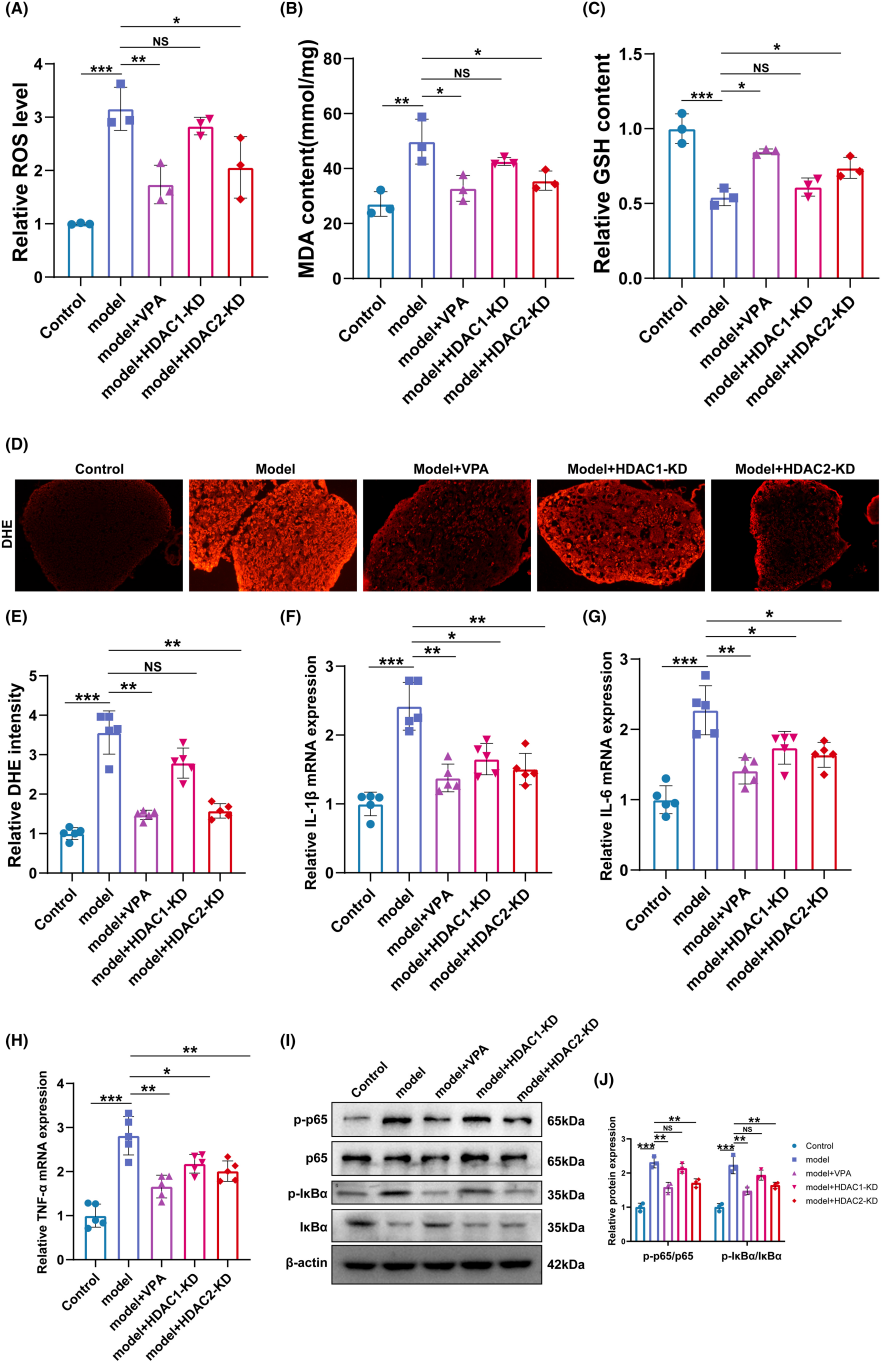
VPA treatment and HDAC2‐KD improved cauda equina injury by suppressing oxidative stress and NF‐κB‐mediated inflammation. (A–C) ROS level, MDA content, and GSH content of different groups were assessed in different groups were assessed by Elisa assay (*N* = 5). (D and E) DHE staining and semi‐quantifications in different groups (*N* = 5). ns = no significant, **p < 0.01; ***p < 0.001. (F–H) The mRNA level of IL‐1β, IL‐6, and TNF‐α in different groups was assessed by qRT‐PCR. (*N* = 5). *p < 0.05; **p < 0.01; ***p < 0.001. (I and J) Western blotting and semi‐quantifications for p‐P65, P65, p‐IκB, and IκB protein in different groups (*N* = 5). **p < 0.01; ***p < 0.001.

To further confirm the demyelination of cauda equina, the sections of each group were incubated with an antimyelin basic protein (MBP) antibody. As shown in Figure 3C,D, compared with the model group, VPA and the knockdown of HDAC2 significantly increased the MBP‐positive staining. Meanwhile, there were no remarkable differences in the fluorescence intensity of MBP between HDAC1 and model groups. Taken together, these results suggested that the protective effect of VPA on CEI may mainly depend on the inhibition of HDAC2 rather than HDAC1.

### 3.3 VPA treatment and HDAC2‐KD improved CEI by suppressing oxidative stress and inflammation

A previous study suggested that oxidative stress plays an important role in the pathological process of CEI.^27^ Liao et al reported that VPA prevents radiation‐induced injury in hippocampal neurons by suppressing oxidative stress.^28^ To investigate whether the protective effects of VPA and HDAC2‐KD on cauda equina injury also rely on oxidative stress, the ROS and MDA content were assessed in the homogenate of cauda equina tissue. As shown in Figure 3A,B, the ROS level and MDA content were significantly increased in the CEC model group compared with the control group, while the effect was blocked by VPA treatment and HDAC2‐KD but not by HDAC1‐KD. Moreover, GSH content was also detected in the homogenate of cauda equina tissue. Figure 3C showed that the GSH content was significantly decreased in the CEC model group compared with the control group, while the effect was restored by VPA treatment and HDAC2‐KD but not by HDAC1‐KD.

To further confirm that the protective function of VPA and HDAC2‐KD on cauda equina injury is related to the amelioration of oxidative stress, DHE fluorescence staining of cauda equina tissue was performed (Figure 3D). Consistent with the previous data, VPA and HDAC2‐KD, but not HDAC1‐KD, alleviated the CEC‐induced ROS production.

Previous studies suggested that inflammation contributed to the development of peripheral nerve injury,^29^, ^30^ and HDAC2 inhibited the activation of NF‐κB.^31^, ^32^ To this end, we analyzed whether VPA and HDAC2‐KD improved cauda equina injury by inhibiting NF‐κB‐mediated inflammation. The expression of pro‐inflammation factors IL‐1β, TNF‐α, and IL‐6 was assessed by qRT‐PCR. As shown in Figure 3F–H, the expression levels of IL‐1β, TNF‐α, and IL‐6 were significantly increased in the CEC model group in comparison with the control group, while the effect was blocked by VPA treatment, HDAC1‐KD, and HDAC2‐KD. Moreover, the activation of NF‐κB was assessed by western blot. The results showed that the levels of p‐p65 and p‐IκBα were remarkably increased in the CEC model group, and the increased levels of p‐p65 and p‐IκBα were significantly lowered after the treatment of VPA or HDAC2‐KD. The above results indicated that the NF‐κB was activated in the CEC model and can be blocked by VPA or HDAC2‐KD. In addition, that the results showed that although both HDAC1‐KD and HDAC2‐KD can inhibit the activation of NF‐κB, HDAC2‐KD showed higher efficiency. Resultantly, our data indicated that VPA and HDAC2‐KD improved the CEI by suppressing oxidative stress and inhibiting NF‐κB‐mediated inflammation.

### 3.4 VPA and HDAC2‐KD improved the CEI by regulating ferroptosis

**FIGURE 4:**
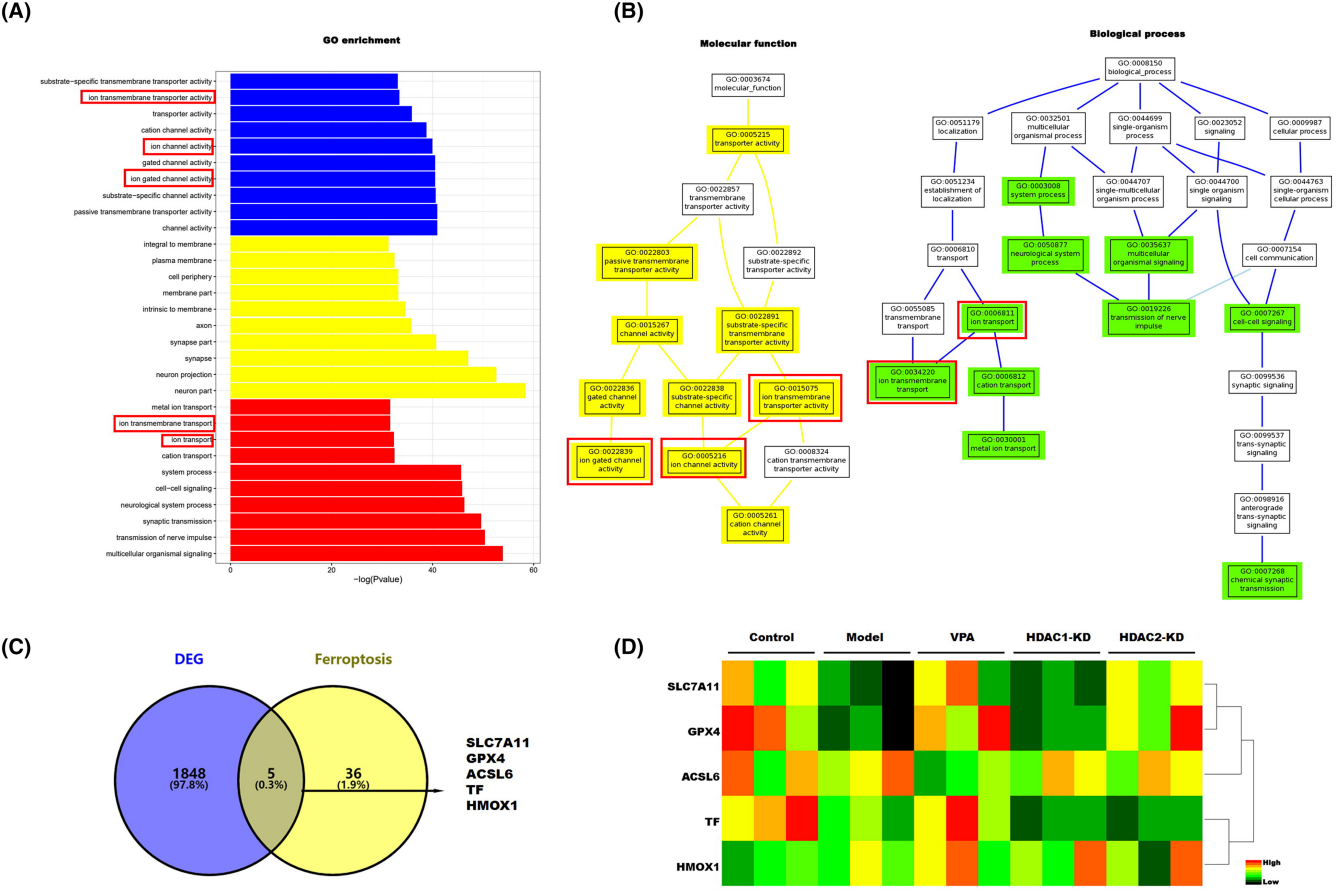
Ferroptosis contributes to the cauda equina injury. (A) GO enrichment of different expression genes in the CEC model with or without VPA treatment. (B) Molecular function and biological process of different expression genes in the CEC model with or without VPA treatment. (C) The ferroptosis genes in DEGs were selected by Venn analysis. (D) The SLC7A11, GPX4, ACSL6, TF, and HMOX1 in different groups were assessed by qRT‐PCR array.

To clarify the underlying mechanism of VPA on CEI protection, we extracted the total RNA of cauda equina tissue of the CEC model with or without VPA treatment for RNA‐sequencing (RNA‐seq). A total of 1853 differentially expressed genes (DEGs) were identified between the model and VPA groups (*p* < 0.05) (Table S1). Functional enrichment analysis by Gene Ontology (GO) showed that ion‐related functions were significantly enriched (Figure 4A,B). Helgudottir et al. reported that Fe^2+^ iron export from brain capillary endothelial cells was elevated after treatment with VPA.^33^ Ounjaijean et al. found that VPA treatment in patients with epilepsy contributed to the metabolism of iron.^34^ In addition, iron and ROS are increasingly recognized as important initiators and mediators of cell death in a variety of organisms and pathological situations.^35^ Therefore, we hypothesized that VPA may improve the CEI by regulating ferroptosis. A Venn diagram analysis identified five common genes (SLC7A11, TF, ACSL6, GPX4, and HMOX1) between ferroptosis‐related genes and DEGs (Figrue 4 C). The expression of these five common genes was assessed by RT‐PCR array in the control group and CEC model with or without VPA, HDAC1‐KD, and HDAC2‐KD. As shown in Figure 4D, we found that SLC7A11 and GPX4, two important ferroptosis suppressor genes, were significantly decreased in the CEC model group, while the expression of SLC7A11 and GPX4 was restored in VPA and HDAC2‐KD groups but not in the HDAC1‐KD group. There was no significant change in the expression of TF, ACSL6, and HMOX1 in each group.

**FIGURE 5:**
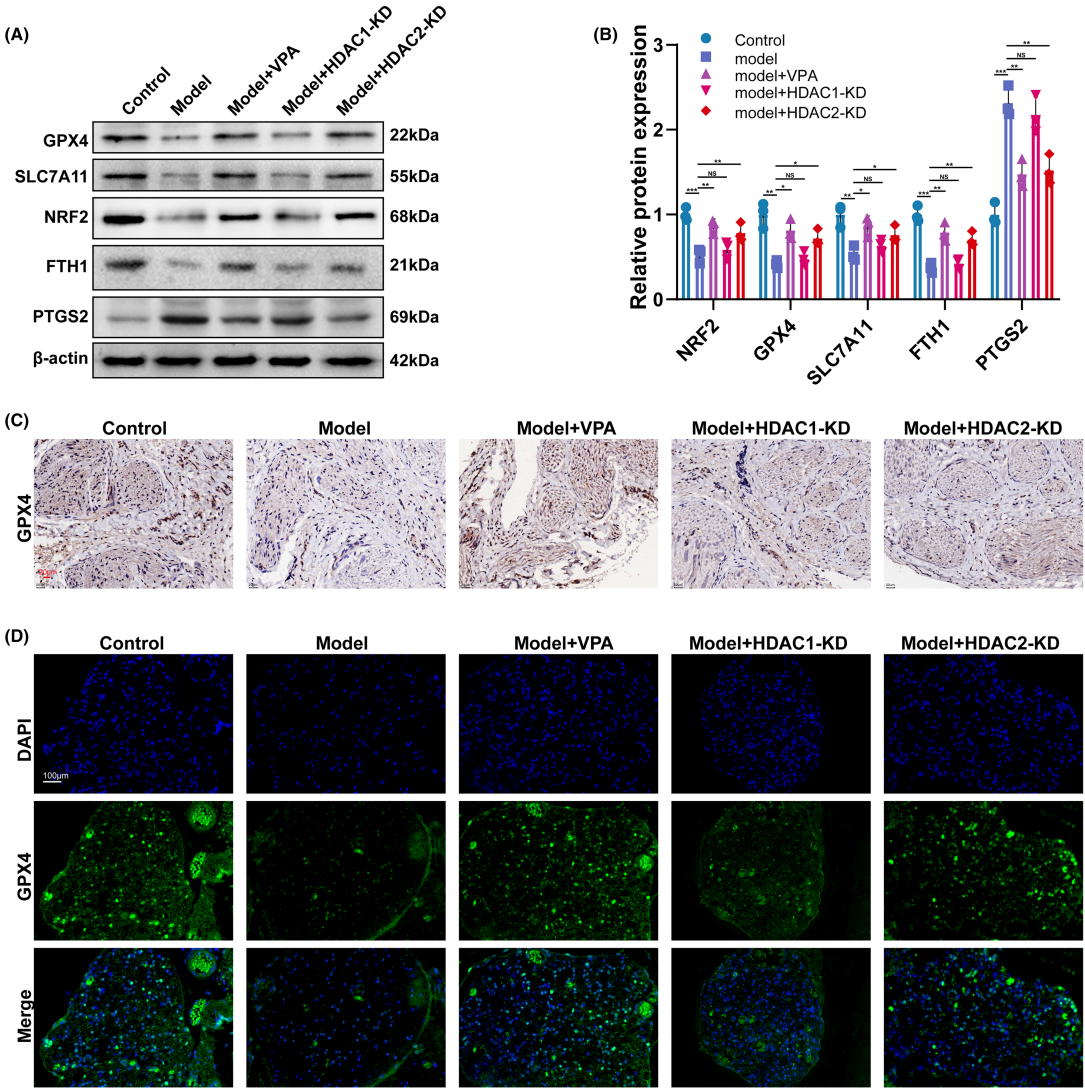
VPA improved the cauda equina injury depending on HDAC2‐mediated ferroptosis. (A and B) Western blotting and semi‐quantifications for GPX4, SLC7A11, Nrf2, FTH1, and PTGS2 protein in different groups (*N* = 5) ns = no significant *p < 0.05; **p < 0.01; *p < 0.001. (C) The protein level of GPX4 in different groups was assessed by IHC (*N* = 5). (D) The protein level of GPX4 in different groups was assessed by IFC (*N* = 5).

Nrf2, a transcription factor, regulates the transcription of GPX4 and SLC7A11 and plays an important role in suppressing lipid peroxidation, which is closely related to the process of ferroptosis. To further confirm that ferroptosis is involved in the function of VPA, the protein expression levels of Nrf2, GPX4, SLC7A11, and ferroptosis‐related markers such as FTH1 and PTGS2, were assessed by western blot. As shown in Figure 5A,B, compared with the control group, the expression of GPX4, SLC7A11, FTH1, and Nrf2 in the CEC model group was markedly decreased, while the effect was restored by VPA treatment and HDAC2‐KD but not by HDAC1‐KD. On the contrary, the expression of PTGS2, a positive marker of ferroptosis, was significantly increased in the CEC model group, while the effect was blocked by VPA treatment and HDAC2‐KD. Moreover, the expression of GPX4 was further assessed by IHC and immunofluorescence. Consistent with previous results, the expression of GPX4 was significantly decreased in the CEC model group compared with the control group, while the expression was restored by VPA treatment and HDAC2‐KD, but not by HDAC1‐KD (Figure 5C,D). Collectively, our data indicated that VPA and HDAC2 improve the CEI through regulating ferroptosis.

### 3.5 VPA inhibited ferroptosis via HDAC2‐H4K12ac axis

**FIGURE 6:**
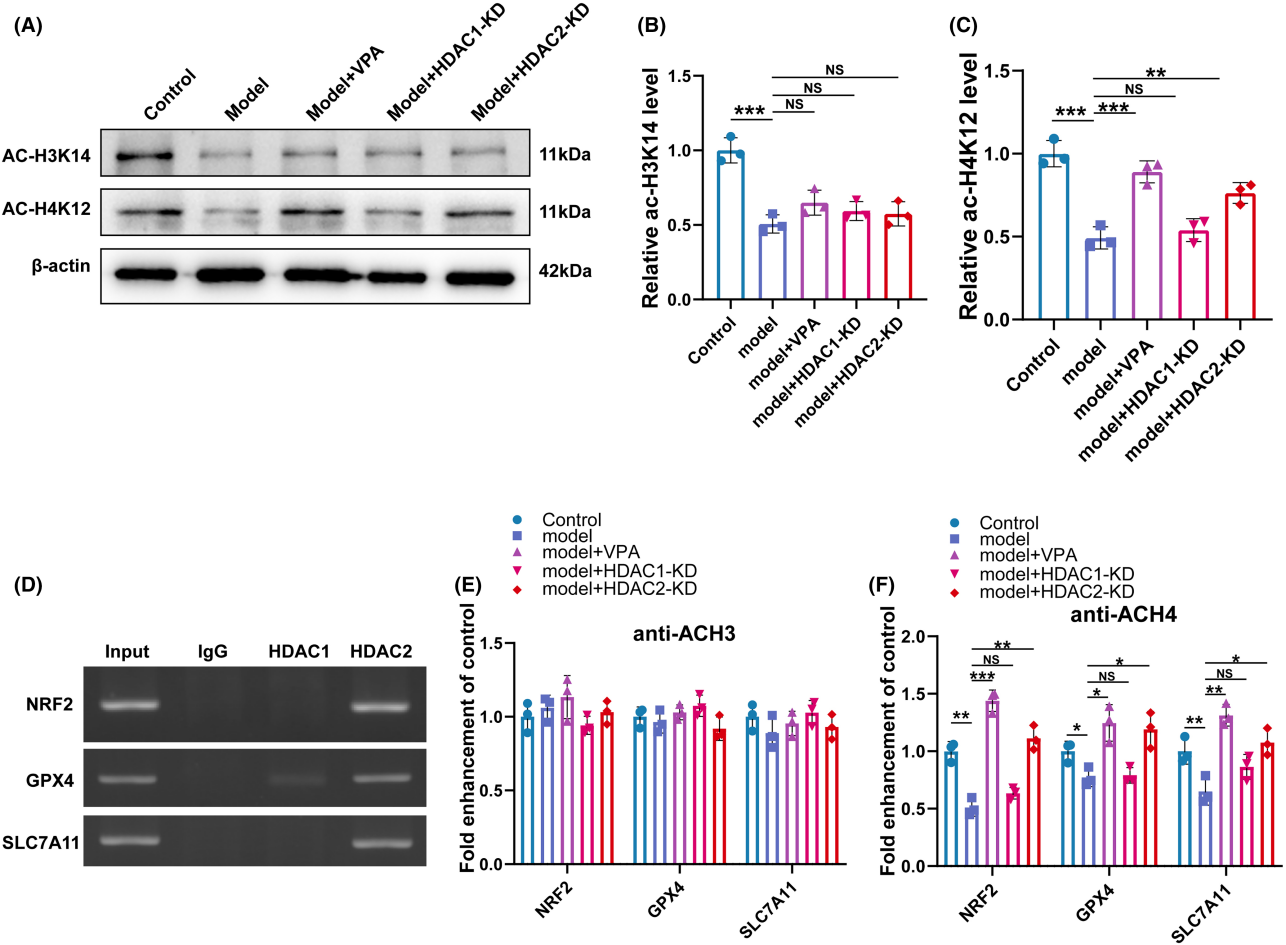
VPA inhibited ferroptosis via HDAC2‐H4K12ac axis. (A‐C) Western blotting and semi‐quantifications for ac‐H3K14 and ac‐H4K12 protein in different groups (*N* = 5) ns = no significant, **p < 0.01; *p < 0.001. (D) ChIP of samples showed specificity of DNA binding for HDAC2 and HDAC1. (E‐F)Changes in histone acetylation in GPX4, SLC7A11, and Nrf2 promoter regions in different groups, sonicated chromatin was IP with anti‐AcH3 or anti‐AcH4 and quantified with qRT‐PCR. ns = no significant *p < 0.05; **p < 0.01; *p < 0.001.

To clarify the epigenetic modulation of ferroptosis‐related gene GPX4, SLC7A11, and Nrf2 by HDAC2‐KD and VPA, the expression level of histone 3 on Lys 14 (H3K14) and histone 4 on Lys 12 (H4K12), the two most abundantly expressed substrates of HDACs, was analyzed by western blot. As shown in Figure 6A,B, we found that the acetylation of H3K14 was significantly decreased in the CEC model group compared with the control group, while the effect was not restored by VPA treatment, HDAC1‐KD, or HDAC2‐KD. Moreover, the level of acetylated H4K12 was also significantly decreased in the CEC model group compared with the control group, but the effect was restored by VPA treatment and HDAC2‐KD but not by HDAC1‐KD (Figure 6A,C).

It was well known that HDACs regulate genes by repressing gene expression through chromatin remodeling. To this end, chromatin immunoprecipitation (ChIP) was performed to confirm whether HDAC1 and HDAC2 were associated with ferroptosis. As shown in Figure 6D, we observed an enrichment of HDAC2, but not HDAC1, at the promoters of GPX4, SLC7A11, and Nrf2. Furthermore, we determined that levels of AcH4, but not AcH3, were increased in the promoters of GPX4, SLC7A11, and Nrf2 in the VPA and the HDAC2‐KD groups in comparison with the CEC model (Figure 6E,F). Taken together, these experiments suggested that VPA treatment and HDAC2‐KD accelerated the expression of the ferroptosis‐related gene GPX4, SLC7A11, and Nrf2 via H4K12ac epigenetic pathways.

### 3.6 HDAC2 facilitated the ferroptosis of DRG in vitro

**FIGURE 7:**
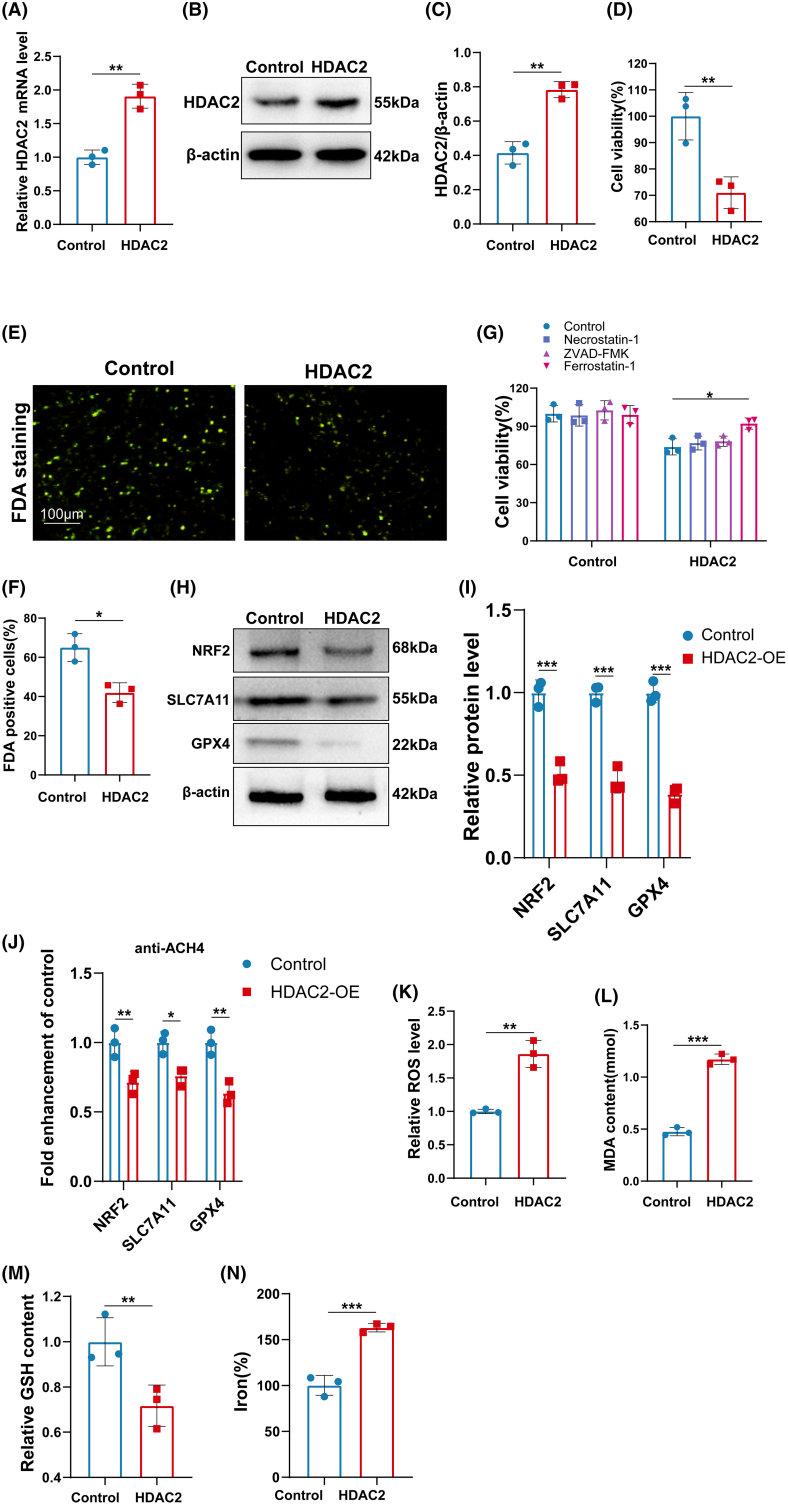
HDAC2 facilitated the ferroptosis of DRG in vitro. (A) The mRNA of HDAC2 was assessed by qRT‐PCR. **p < 0.01. (B and C) Western blotting and semi‐quantifications for HDAC2 in DRG cells with or without HDAC2‐OE. **p < 0.01. (D) DRG cell viability was assessed by CCK8. **p < 0.01. (E and F) DRG cell viability was assessed by FDA staining. *p < 0.05. (G) DRG cell viability treated 1 μM of Ferrostatin‐1, 10 μM of ZVAD‐FMK, or 10 μM of necrostatin‐1 was assessed by CCK8. *p < 0.05. (H and I) Western blotting and semi‐quantifications for NRF2, SLC7A11, and GPX4 in DRG cells with or without HDAC2‐OE. ***p < 0.001. (J) Changes in histone acetylation in GPX4, SLC7A11, and Nrf2 promoter regions DRG cells with or without HDAC2‐OE, sonicated chromatin was IP anti‐AcH4 and quantified with qRT‐PCR. *p < 0.05; **p < 0.01. (K–N) ROS level, MDA content, GSH content, and iron‐level DRG cell with or without HDAC2‐OE were assessed by Elisa assay. **p < 0.01; ***p < 0.001.

To investigate the functions of HDAC2 in the regulation of ferroptosis in DRG neurons. DRG neurons were transfected with lentivirus HDAC2. As shown in Figure 7A–C, the expression of HDAC2 was significantly increased by lentivirus HDAC2 plasmid. The cell viability was analyzed by CCK‐8 assay in the DRG neurons with or without HDAC2 overexpression. As shown in Figure 7D, HDAC2‐OE suppressed the cell viability of DRG neurons. The results from FDA staining further confirmed that HDAC2‐OE inhibited the cell viability of DRG neurons (Figure 7E,F). Interestingly, the inhibitory effect of HDAC2‐OE on DRG neurons cell viability was blocked by ferrostatin‐1 (a specific inhibitor of ferroptosis), but not by ZVAD‐FMK (a specific inhibitor of apoptosis) or necrostatin‐1 (a specific inhibitor of necroptosis) (Figure 7G), indicating that HDAC2 promoted the ferroptosis of DRG neurons.

To confirm the function of HDAC2 in the ferroptosis of DRG neurons, the expression of GPX4, SLC7A11, and Nrf2 was assessed by western blot. Figure 7H,I shows that the expression of GPX4, SLC7A11, and Nrf2 was markedly decreased by HDAC2 overexpression. In addition, the levels of AcH4 were significantly decreased in the promoters of GPX4, SLC7A11, and Nrf2 in HDAC2‐OE DRG neurons (Figure 7J). Moreover, the ferroptosis‐related markers were assessed in HDAC2‐OE DRG neurons. As shown in Figure 7K–N, the ROS level, MDA content, and iron level were significantly increased, while the GSH content was significantly decreased by HDAC2 overexpression, suggesting that HDAC2 promoted the ferroptosis of DRG neurons.

**FIGURE 8:**
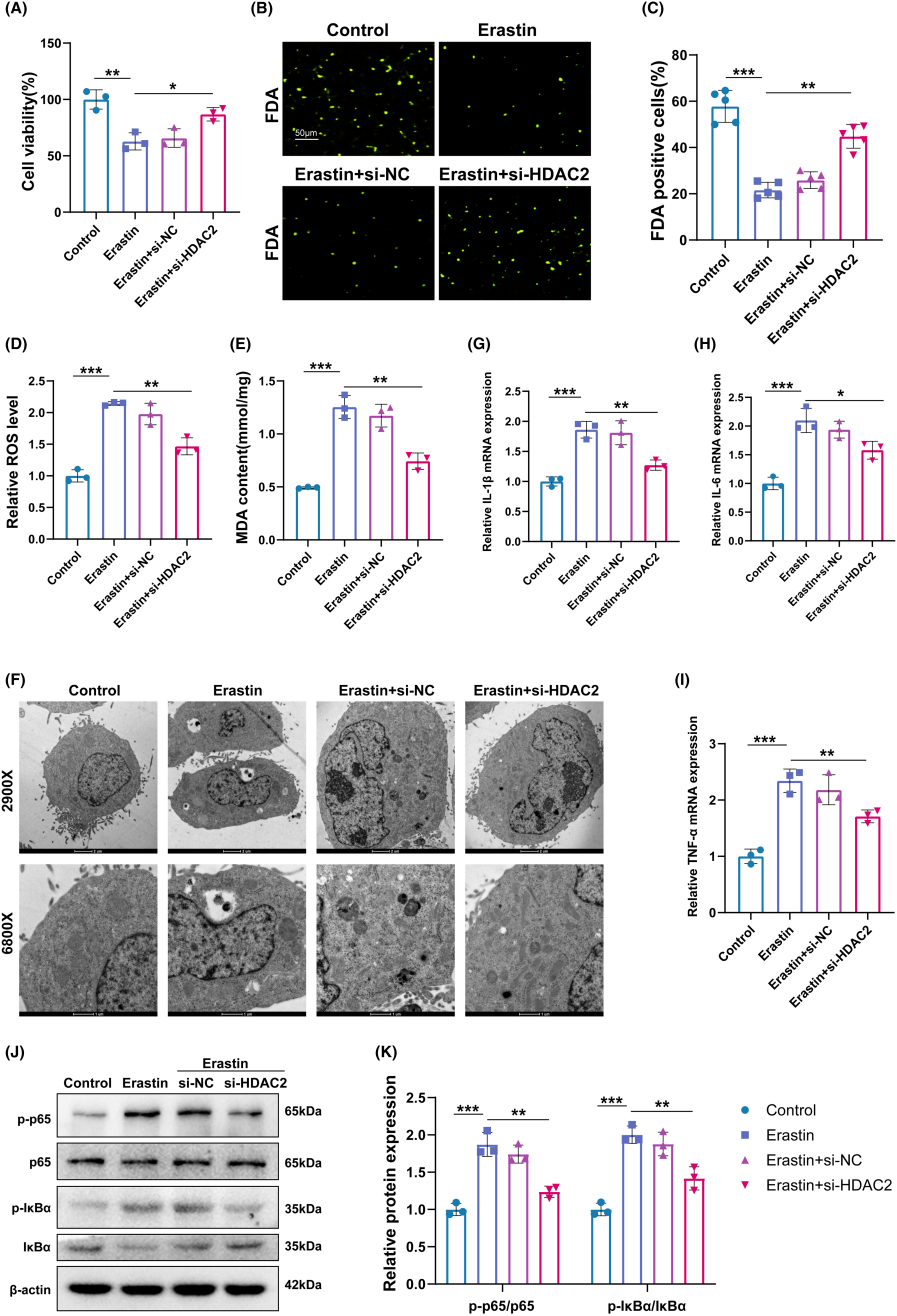
HDAC2 inhibition suppressed NF‐κB‐mediated inflammation and ferroptosis of DRG in vitro. (A) Cell viability of DRG treated with Erastin and HDAC2‐KD or without HDAC2‐KD was assessed by CCK8. *p < 0.05; **p < 0.01. (B and C) Cell viability of DRG treated with Erastin and HDAC2‐KD or without HDAC2‐KD was assessed by FDA staining. *p < 0.01; **p < 0.001. (D and E) ROS level, MDA content of DRG treated with Erastin, and HDAC2‐KD or without HDAC2‐KD were assessed by Elisa. *p < 0.01; **p < 0.001. (F) Ferroptosis of DRG treated with Erastin and HDAC2‐KD or without HDAC2‐KD was assessed by TEM. The arrows indicated the morphological changes. (G‐I) The mRNA level of IL‐1β, IL‐6, and TNF‐α in different groups was assessed by qRT‐PCR. *p < 0.05; **p < 0.01; ***p < 0.001. (J–K) Western blotting and semi‐quantifications for p‐P65, P65, p‐IκB, and IκB protein in different groups. *p < 0.05; **p < 0.01; ***p < 0.001.

To further confirm the function of HDAC2 on DRG ferroptosis. DRG neurons were treated by Erastin with or without HDAC2‐KD. As shown in Figure 8A, the cell viability of DRG neurons was significantly decreased by Erastin, while the effect was reversed by HDAC2‐KD. The result was further confirmed by FDA staining (Figure 8B,C). Next, the ROS level and MDA content were assessed by Elisa, Figure 8D,E indicated that si‐HDAC2 prevented the increase of ROS and MDA induced by Erastin in DRG neurons. It was reported that the morphology of mitochondrial ultrastructure in cells with ferroptosis was altered.^36^ Transmission electron microscope analysis demonstrated that mitochondria in Erastin‐stimulated DRG exhibited a reduced volume, increased electron density, and damaged mitochondrial cristae, while HDAC2‐KD alleviated these detrimental morphological changes (Figure 8F). In addition, the HDAC2‐KD inhibited the production of IL‐1β, IL‐6, and TNF‐α (Figure 8G–I). The level of p‐p65 was significantly raised after the treatment of Erastin (Figure 8J), and the result indicated that the NF‐κB pathway was associated with ferroptosis. What is more, the increased levels of p‐p65 and p‐IκBα induced by Erastin were decreased by the treatment of HDAC2‐KD, indicating that the activation of NF‐κB signaling induced by Erastin was suppressed by HDAC2‐KD (Figure 8J,K). Taken together, our data suggested that HDAC2 functions as a pro‐ferroptosis and pro‐neuroinflammatory gene in the DRG neurons.

## 4 DISCUSSION

The pathogenesis of CEI is complex and there is no effective treatment strategy currently. Our previous study confirmed that VPA has neuroprotective effects on the CEI rat model.^7^ However, the underlying mechanism remains to be fully elucidated. Previous studies have confirmed that HDAC1 and HDAC2 are the main targets of VPA. Jie Sun et al. reported that VPA suppressed cell proliferation by targeting HDAC1/2 and HDAC1/PTEN/Akt signaling.^37^ In this present study, HADC1 and HDAC2 expression were significantly increased in the CEC model, which could be partly restored by VPA treatment in DRG and cauda equina, indicating the histone acetylation homeostasis imbalance in the pathological process of cauda equina injury. HDAC1 and HDAC2 are upregulated in many diseases and contribute to the development of these diseases by regulating aberrant pathways of cells.^38^ Valérie Brügger et al. reported that early response to nerve injury was controlled by histone deacetylase 2 (HDAC2), and HDAC1/2 inhibitor treatment early after lesion accelerated functional recovery and enhanced regeneration.^39^ The findings of this present study demonstrated that VPA improves the function of CEI by inhibiting HDAC1 and HDAC2.

To clarify whether the VPA improves CEI through regulating the expression of HDAC1 or HDAC2, the expression of HDAC1 and HDAC2 was knocked down by adenoviral transduction. One week after cauda equina injection of adenoviral sh‐HDAC1 and sh‐HDAC2, the expression of HDAC1 and HDCA2 in the DRG and cauda equina was significantly decreased. Consistent with our previous results, the pathological results showed VPA could alleviate the Wallerian degeneration and demyelination of nerve fibers after cauda equina injury and promote axonal growth. Interestingly, we found the same results only in the HDAC2‐KD group, but not in the HDAC1‐KD group, suggesting that VPA's treatment efficacy in CEI may depend on HDAC2. The results above were consistent with behavioral results, in which the BBB scores and withdrawal thresholds showed that the VPA and HDAC2‐KD groups, but not the HDAC1‐KD, alleviated motor and sensory dysfunction. Another study also showed that HDAC2 but not HDAC1 negatively regulates memory formation and synaptic plasticity,^26^ which is consistent with our results. Thus, the treatment of VPA in CEI mainly depends on HDAC2 rather than HDAC1.

After central/peripheral nerve injury, a large number of infiltrated inflammatory cells and the soluble pro‐inflammatory mediators in the center of the injury cause secondary inflammatory damage. The production of ROS during acute and chronic neuroinflammation damages neural cells. Liu et al. suggested that oxidative stress was an independent risk factor for cauda equina syndrome and the oxidative stress marker MDA was significantly increased in CEI rats.^27^ In this current study, we also found that oxidative stress was markedly increased in the CEC rat model, which could be blocked by the administration of VPA. The same effect was observed in the CEC rat model with HDAC2‐KD, suggesting that the effect of VPA on improving CEI may be dependent on HDAC2‐mediated oxidative stress, which is in accord with previous reports.^40^ In addition, we revealed that NF‐κB pathway‐mediated inflammation was upregulated in the CEC model, whereas VPA, HDAC1‐KD, and HDAC2‐KD inhibited the activation of the NF‐κB pathway, which also is in line with previous studies.^41^, ^42^ The data above suggested that VPA improvement in CEI may also depend on NF‐κB pathway‐mediated inflammation.

To further clarify the mechanism of VPA improving CEI, a high‐throughput sequence was performed in the CEC model with or without VPA treatment. As we know, the imbalance of ferric ions induces intracellular ROS generation via Fenton reactions. Although ROS is necessary for physiological signal pathways, oxidative stress contributes to lots of tissue damage and diseases. Moreover, oxidative stress can cause ferroptosis, an iron‐dependent type of cell death. The study regarding the crosstalk between oxidative stress and ferroptosis has been focused in recent years. Growing evidence has suggested that ferroptosis plays an important role in the onset and exacerbation of spinal cord injury. Xue Yao et al. reported that deferoxamine promoted recovery of traumatic spinal cord injury by inhibiting ferroptosis.^43^ Ming‐Hao Ge et al. reported that Zinc attenuated ferroptosis and promoted functional recovery in contusion spinal cord injury by activating the Nrf2/GPX4 defense pathway.^44^ In addition, ferroptosis is also an important inducer of inflammation.^36^, ^45^ In this present study, GO analysis found that differentially expressed genes (DEG) were enriched in the ion‐related pathway (ion transmembrane transporter activity, ion channel activity, ion gated channel activity, ion transport). Consistently, our data demonstrated that the expression of anti‐ferroptosis genes including Nrf2, GPX4, FTH1, and SLC7A11 was downregulated and ferroptosis marker PTGS2 was upregulated in the CEC group, whereas the effect was restored by VPA treatment and HDAC2‐KD, suggesting that VPA improved CEI by regulating HDAC2‐mediated ferroptosis.

Although the function of HDAC2 in the production of ROS has been widely reported,^46^ the regulation of ferroptosis by HDAC2 has not been reported. Therefore, the function of HDAC2 on the ferroptosis of DRG was further confirmed by in vitro experiments in this present study. We found that overexpression of HDAC2 significantly increased the expression levels of ferroptosis‐associated indicators in DRG neurons, as evidenced by the upregulation of oxidative stress and downregulation of Nrf2, GPX4, and SLC7A11. Moreover, HDAC2‐KD significantly inhibited the ferroptosis, ROS, and inflammation induced by Erastin. These results further confirm our conclusion that VPA improves CEI by regulating HDAC2‐mediated ferroptosis.

In the present study, acetylation of H3, H3K14, and acetylation of H4, H4K12, were significantly decreased in the CEC model. Furthermore, the acetylation of H4, H4K12, could be restored by VPA and HDAC2‐KD, but cannot by HDAC1‐KD. Our findings are consistent with previous studies, which demonstrated increased acetylation of histones H4K12 in mouse forebrain primary neuronal cultures following treatment with HDAC2 inhibitors.^47^ In addition, ChIP experiments in our study demonstrated that HDAC2 bound to GPX4, SLC7A11, and Nrf2 endogenous promoters. In line with the results, the level of AcH4, but not AcH3, was increased in the promoters of ferroptosis‐related genes in the VPA and HDAC2‐KD group, suggesting that HDAC2‐mediated ferroptosis by inhibiting the acetylation of H4K12. This conclusion was further verified by the in vitro experiment. HDAC2 overexpression significantly decreased the level of Ac‐H4 in the promoters of ferroptosis‐related genes, including Nrf2, GPX4, and SLC7A11, indicating that HDAC2 regulated the transcription of ferroptosis‐related genes. Taken our data together, we found that VPA and HDAC2‐KD inhibited neuronal ferroptosis by regulating the histone acetylation levels of GPX4, SLC7A11, and Nrf2 promoter regions and promoting the expression of GPX4, SLC7A11, and Nrf2.

Although we found that VPA and HDAC2 could improve motor and sensory functions after CEI, there were still some deficiencies in this study. For example, some symptoms of cauda equina injury were not detected, such as sensory disorders in the sellar area and sphincter dysfunction. In addition, gender was not considered in the evaluation of hyperalgesia. In the mechanistic research section, we explored HDAC1 and HDAC2 as the major targets of VPA for improving CEI. However, we cannot exclude the possibility that other HDAC family members also play a role in recovery from CEI. These deficiencies will be addressed in future research.

In conclusion, we demonstrated that VPA improved the CEI by inhibiting ferroptosis via the HDAC2–H4K12AC axis. This study provides a new idea for the treatment of clinical CES. Firstly, we confirmed the function of histone acetylation homeostasis imbalance in the pathological process of CEI and provided a new direction for the treatment of CES from epigenetic regulation and histone acetylation modification. Second, the key role of HDAC2 in the repair and prognosis of CEI was confirmed, thereby laying the experimental foundation for the future clinical development of new drugs. Finally, we elucidated the molecular mechanism of ferroptosis in the pathology of CEI, which may be one of the reasons for the difficulty of repair after CES. Taken together, this study explained the potential mechanism of VPA in the treatment of CEI and provided new insights for clinical treatment.

## AUTHOR CONTRIBUTION

QJK and JM designed the experiments. QJK, FDL, and KQS carried out the experiments. All authors proofread the manuscript. QJK, FDL, and KQS supervised the experiments and analyzed the results. QJK and XFS wrote the manuscript. All authors contributed to the article and approved the submitted version.

## FUNDING INFORMATION

The study is supported by the National Natural Science Foundation of China (grant no. 81801226).

## CONFLICT OF INTEREST STATEMENT

There are no competing interests.

## CONSENT FOR PUBLICATION

All authors have given their consent for publication.

## Supporting information


Table S1.

## Data Availability

All the data and materials concerned with the manuscript are available with the corresponding author and can thereby asked.
